# Toll-like receptor 4 and lipopolysaccharide from commensal microbes regulate Tembusu virus infection

**DOI:** 10.1016/j.jbc.2022.102699

**Published:** 2022-11-13

**Authors:** Zhen Wu, Tao Hu, Andres Merits, Yu He, Mingshu Wang, Renyong Jia, Dekang Zhu, Mafeng Liu, Xinxin Zhao, Qiao Yang, Ying Wu, Shaqiu Zhang, Juan Huang, Sai Mao, Xumin Ou, Qun Gao, Di Sun, Yunya Liu, Ling Zhang, Yanling Yu, Anchun Cheng, Shun Chen

**Affiliations:** 1 Institute of Preventive Veterinary Medicine, Sichuan Agricultural University, Chengdu, Sichuan, China; 2 Institute of Technology, University of Tartu, Tartu, Estonia; 3 Research Center of Avian Disease, College of Veterinary Medicine, Sichuan Agricultural University, Chengdu, Sichuan, China; 4 Key Laboratory of Animal Disease and Human Health of Sichuan Province, Sichuan Agricultural University, Chengdu, Sichuan, China

**Keywords:** TMUV, virus receptor, TLR4, lipopolysaccharide, Abx, antibiotic, BHK, baby hamster kidney cell, co-IP, coimmunoprecipitation, DENV, dengue virus, E, envelope protein, hpi, hours postinfection, IAV, influenza A virus, IFA, immunofluorescence assay, IL6, interleukin 6, LBP, LPS-binding protein, LPS, lipopolysaccharide, Nluc, nanoluciferase, qPCR, quantitative PCR, SPF, specific pathogen-free, SRIP, single-round infection virus particle, TCID_50_, 50% tissue culture infectious dose, TLR4, Toll-like receptor 4, TMUV, Tembusu virus

## Abstract

Unlike most flaviviruses transmitted by arthropods, Tembusu virus (TMUV) is still active during winter and causes outbreaks in some areas, indicating vector-independent spread of the virus. Gastrointestinal transmission might be one of the possible routes of vector-free transmission, which also means that the virus has to interact with more intestinal bacteria. Here, we found evidence that TMUV indeed can transmit through the digestive tract. Interestingly, using an established TMUV disease model by oral gavage combined with an antibiotic treatment, we revealed that a decrease in intestinal bacteria significantly reduced local TMUV proliferation in the intestine, revealing that the bacterial microbiome is important in TMUV infection. We found that lipopolysaccharide (LPS) present in the outer membrane of Gram-negative bacteria enhanced TMUV proliferation by promoting its attachment. Toll-like receptor 4 (TLR4), a cell surface receptor, can transmit signal from LPS. We confirmed colocalization of TLR4 with TMUV envelope (E) protein as well as their interaction in infected cells. Coherently, TMUV infection of susceptible cells was inhibited by an anti-TLR4 antibody, purified soluble TLR4 protein, and knockdown of TLR4 expression. LPS-enhanced TMUV proliferation could also be blocked by a TLR4 inhibitor. Meanwhile, pretreatment of duck primary cells with TMUV significantly impaired LPS-induced interleukin 6 production. Collectively, our study provides first insights into vector-free transmission mechanisms of flaviviruses.

Egg drop disease syndrome, which is caused by Tembusu virus (TMUV), has emerged as a major avian health problem in Asia during the last decade (1, 2). The clinical symptoms of the disease include weight loss and egg production decline. The later stage of the disease is characterized by neurological symptoms, paralysis, and unstable walking. In addition, some sick birds exhibit additional symptoms, such as increased body temperature and discharge of green stool (1). TMUV is a flavivirus that has not been reported to cause symptoms in humans; however, the virus and neutralizing antibodies of TMUV can be detected in the blood of works of duck farms (3). Serum neutralizing antibodies can also be detected in people with no connection to duck factories (4) indicating that TMUV also represents the public health risk.

Under natural conditions, pathogenic flaviviruses are mainly transmitted by arthropods, such as mosquitoes and ticks. However, animals are still infected with TMUV during the cold season, that is, the prevalence of TMUV does not correlate with seasonal temperature changes that are not consistent with prevalence of mosquito-transmitted flaviviruses (5). Previous research has suggested vector-less transmission for Japanese encephalitis virus and West Nile virus (6, 7). Coherently, Li *et al.* (5) found that both direct contact and aerosol transmission can mediate the spread of TMUV among ducks. TMUV transmission through airborne route and direct contact implies that compared with mosquito transmission the virus and commensal microbes have more interplay in the respiratory and digestive tracts during infection. The interaction between intestinal commensal bacteria and enteric viruses has been extensively studied and shown to promote the proliferation of different enteroviruses (8, 9). Virions are stabilized by the presence of bacteria upon heat treatment (10). The interaction between mouse mammary tumour virus and microbiota induces an immune evasion pathway (8). It has been also noticed that commensal microbiota regulate immunity in the respiratory mucosa after infection with influenza A virus (IAV) and that commensal microbiota–induced immunity can inhibit IAV replication (11). The direct interaction between IAV and commensal microbiota products, such as lipopolysaccharides (LPSs), has also been shown to alter the morphology of IAV virions (12).

Toll-like receptors (TLRs) are innate immune receptors that are related to the Toll protein of *Drosophila* (13). TLR4 was the first TLR found in humans and is expressed in almost all cell types (14). Several viral proteins have previously been reported to activate the TLR4 receptor, including G protein of vesicular stomatitis virus, F protein of respiratory syncytial virus, glycoprotein of Ebola virus, and NS1 protein of dengue virus (DENV) (15, 16). TLR4 and its ligand incorporated into the retroviral envelope augment virus transmission (17). However, the mechanism(s) by which TLR4 interacts and uses flavivirus components has never been reported.

Here, we examined the mechanisms of LPS and TLR4-mediated increase of TMUV infectivity. For the first time, it was shown that TLR4 plays an important role during attachment step of TMUV infection. LPS was also found to be involved in TLR4-mediated enhancement of TMUV infection, and the knockdown of TLR4 expression was shown to result in reduced ability of LPS to promote virus adsorption. We propose a model that can explain the complex and important role played by LPS and TLR4 in TMUV infection: (1) TMUV binds LPS receptors by competing with LPS, thereby inhibiting host inflammatory signal transduction; (2) LPS also promotes TLR4-mediated TMUV attachment. As a consequence, reduced LPS sources such as intestinal commensal bacteria decrease TMUV proliferation *in vivo*. Importantly, these findings about the novel role of LPS in TMUV infection may apply to flavivirus infection in general.

## Results

### Intestinal bacteria facilitate TMUV oral infection

**Figure 1:**
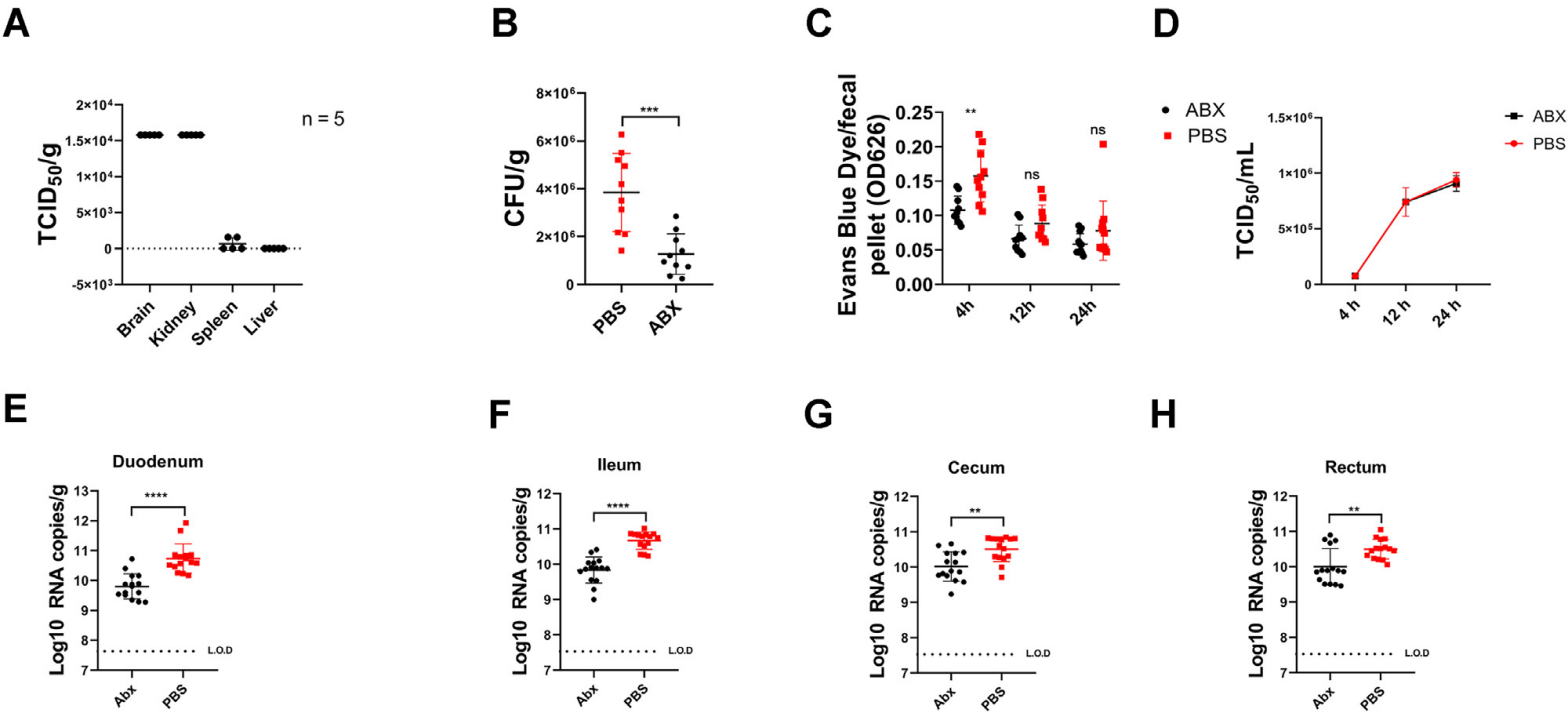
**Intestinal bacteria facilitate TMUV infection.***A*, 2-day-old ducklings (n = 5) were gavaged with 1 × 10^6^ TCID_50_ of TMUV for 24 h. The brain, kidneys, spleen, and liver were collected for virus detection. Virus titers were determined in BHK-21 cells. *B*, the ducks were treated with PBS or Abx for 20 days (n = 5) for each group. The feces were plated and grown anaerobically and aerobically. Resulting colony-forming units (CFUs) per gram of feces are blotted for each group. *C*, untreated or Abx-treated ducklings (n = 5 for each group) were orally administered Evan’s blue dye, and feces were collected at 4, 12, and 24 h post-treatment. Feces were suspended in PBS, and the amount of dye excreted was determined. *D*, TMUV (5 × 10^5^ TCID_50_) was incubated with Abx (0.5 mg/ml) for 1 h at 4 °C, after which it was used to infect duck primary cells. Supernatants were sampled at 4, 12, and 24 hpi, and TMUV titers were analyzed using limiting dilution method. *E*–*H*, groups of PBS-treated (*red*) and Abx-treated (*black*) ducks (n = 5 ducks) were orally infected with 1 × 10^6^ TCID_50_ units of TMUV. At 24 h, the virus genomes present in the duodenum (*E*), ileum (*F*), cecum (*G*), and rectum (*H*) were quantified by using an RT–qPCR assay. *Horizontal dotted lines* denote the limit of detection (LOD) of the assay. The data are the mean ± SD of three independent experiments. ∗*p* < 0.05; ∗∗*p* < 0.001; ∗∗∗*p* < 0.001; and ns. Abx, antibiotic; BHK, baby hamster kidney cell; hpi, hours postinfection; ns, not significant; qPCR, quantitative PCR; TCID_50_, 50% tissue culture infectious dose; TMUV, Tembusu virus.

To explore additional routes of TMUV transmission, 2-day-old ducklings were orally gavaged with 1 × 10^6^ 50% tissue culture infectious dose (TCID_50_) of TMUV. Infectious virus could be detected in the brain and kidney and in trace amounts also in spleen at 24 h postinfection (hpi) (Fig. 1*A*), confirming that TMUV could be infected through the digestive tract. To investigate the potential effect of the intestinal microbiota on TMUV infection, we depleted microbes by treating 1-day-old specific pathogen-free (SPF) ducklings with antibiotics (Abx). As a side effect, the effect of Abx on the intestinal flora could affect the growth of animals (18). To reduce the side effects of Abx treatment, we treated the ducks gradually increasing the concentration of the Abx. Using this protocol, Abx treatment did not affect the growth of the ducks. On the 20th day, it was confirmed that the Abx treatment significantly reduced the cultural intestinal bacteria (Fig. 1*B*). We also monitored the intestinal movement of the ducks. In Abx-treated ducks, the dye transit was significantly delayed at the early stage (4 h post-treatment) but returned to the normal from 12 h post-treatment (Fig. 1*C*). The *in vitro* experiments performed using duck primary cells showed that Abxs did not affect virus replication (Fig. 1*D*). At the same time, Abx treatment reduced TMUV proliferation in infected ducks; in average, nearly 10-fold reduction was observed in the duodenum and threefold reduction in the ileum, cecum, and rectum (Fig. 1, *E*–*H* and Table 1). These data allow to concluding that the depletion of microbiota affected TMUV proliferation upon oral administration.

**Table 1:** RT–qPCR primers used in this study

Gene name	Primer sequences (5′ to 3′)
Duck TLR4	Forward: CCATGGTGCTTTCTCCTGTGT
	Reverse: CTGGGTGGTGTTTGGGACTT
TMUV E	Forward: AATGGCTGTGGCTTGTTTGG
	Reverse: GGGCGTTATCACGAATCTA
Duck IL6	Forward: CCAAGGTGACGGAGGAAGAC
	Reverse: GTGAGGAGGGATTTCTGGGTAG
Duck IFNbeta	Forward: TCTACAGAGCCTTGCCTGCAT
	Reverse: TGTCGGTGTCCAAAAGGATGT
Duck β-actin	Forward: GATCACAGCCCTGGCACC
	Reverse: CGGATTCATCATACTCCTGCTT

### LPS treatment affects TMUV *in vitro* infection

**Figure 2:**
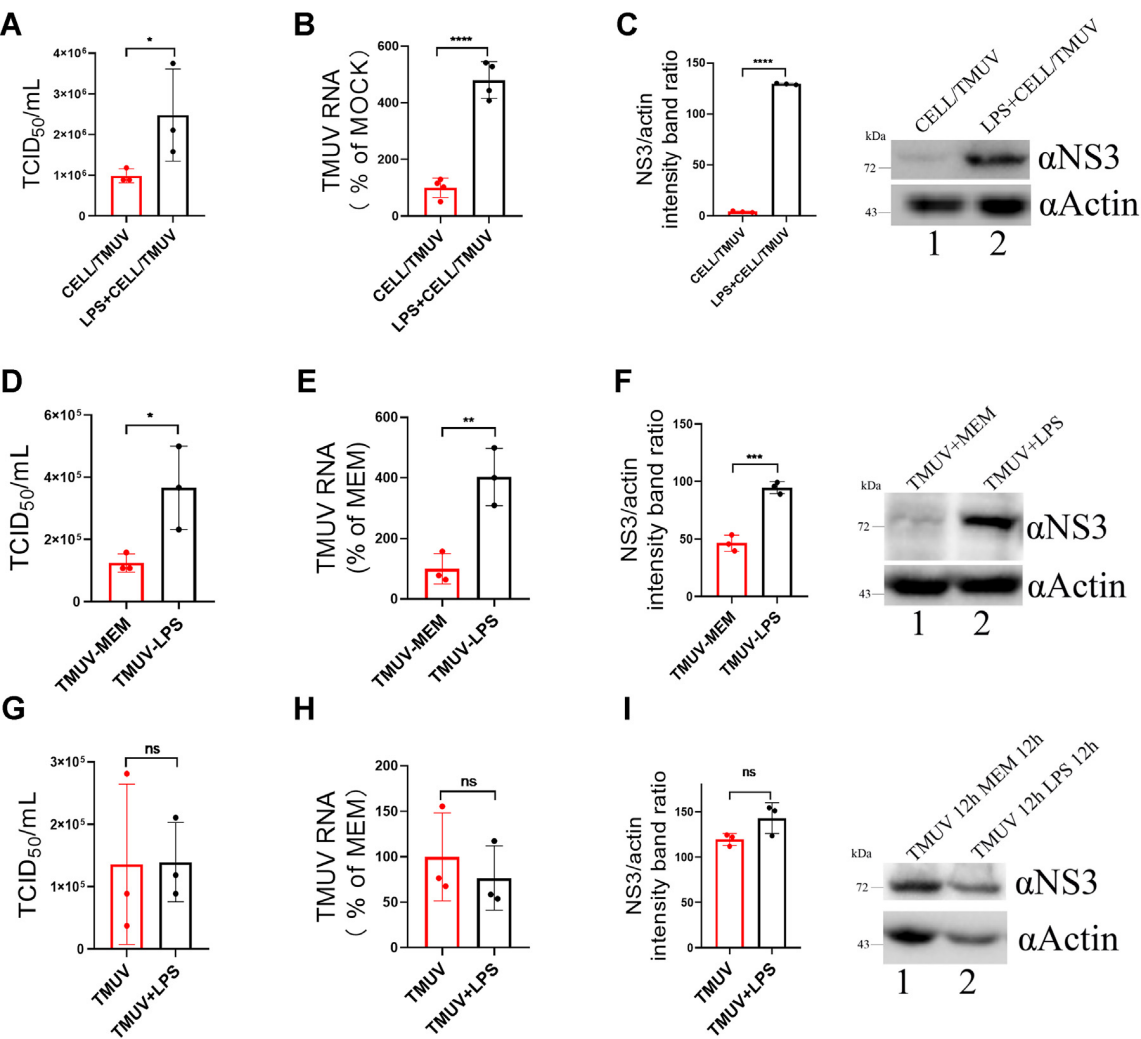
**LPS pretreatment enhances TMUV infection in cell culture.***A*–*C*, the cultures of 1 × 10^6^ duck primary cells were treated with LPS (20 μg; LPS + CELL/TMUV) or mock-treated (CELL/TMUV) for 12 h before infection with 1 × 10^5^ TCID_50_ TMUV. After 24 hpi, the cells and culture supernatants were collected and assayed for extracellular infectious virus production (*A*), intracellular RNA replication; average level of TMUV RNA in the mock-treated cells was taken as 100% (*B*), and NS3 protein expression (*C*). *D*–*F*, 1 × 10^5^ TCID_50_ of TMUV was preincubated with 20 μg of LPS (TMUV-LPS) or MEM (TMUV-MEM) for 1 h at 4 °C and used to infect duck primary cells. About 24 hpi, the culture supernatants and cells were collected and assayed for extracellular infectious virus titers *(D*), intracellular viral RNA amount; average level of TMUV RNA in the cells infected with MEM-treated virus was taken as 100% (*E*) and NS3 protein expression (*F*). *G*–*I*, 1 × 10^6^ duck primary cells were infected with 1 × 10^5^ TCID_50_ TMUV for 12 h and then treated with 20 μg LPS (TMUV + LPS) or mock-treated (TMUV) for 12 h. After this, cell culture supernatants and cells were collected and assayed for infectious virus titers (*G*), intracellular viral RNA amount; average level of TMUV RNA in the mock-treated cells was taken as 100% (*H*) and NS3 protein expression (*I*). The data are the mean ± SD of three experiments. ∗*p* < 0.05; ∗∗*p* < 0.01; ∗∗∗*p* < 0.001; ∗∗∗∗*p* <0.0001; ns. hpi, hours postinfection; LPS, lipopolysaccharide; MEM, minimum essential medium; ns, not significant; TCID_50_, 50% tissue culture infectious dose; TMUV, Tembusu virus.

To explore the mechanism of TMUV infection through intestine, we focused on the LPS, which is the outer membrane major component of Gram-negative bacteria and is known to modulate flavivirus infection (19). It has been reported that DENV infection was inhibited by pretreating monocytes/macrophages with LPS and that this treatment blocked DENV entry (20). In contrast to this report, we observed that the pretreatment of duck primary cells with LPS for 12 h significantly enhanced TMUV infection, resulting in an approximately 60% increase of infectious virus in the supernatant, 4.6-fold increase of TMUV RNA levels in infected cells, and concomitant nearly ninefold increase of the NS3 protein levels (Fig. 2, *A*–*C*). The effects resulting from incubation of TMUV virions and LPS prior were similar, albeit less pronounced: this treatment resulted in a nearly 60% increase of infectious virus in the supernatant, 2.5-fold increase of intracellular TMUV RNA levels, and 2.5-fold increase of the NS3 protein levels (Fig. 2, *D*–*F*). In contrast, when the cells were first infected with TMUV and treated with LPS at 12 h hpi, no significant change in TMUV proliferation, RNA, or NS3 protein levels was observed (Fig. 2, *G*–*I*).

**Figure 3:**
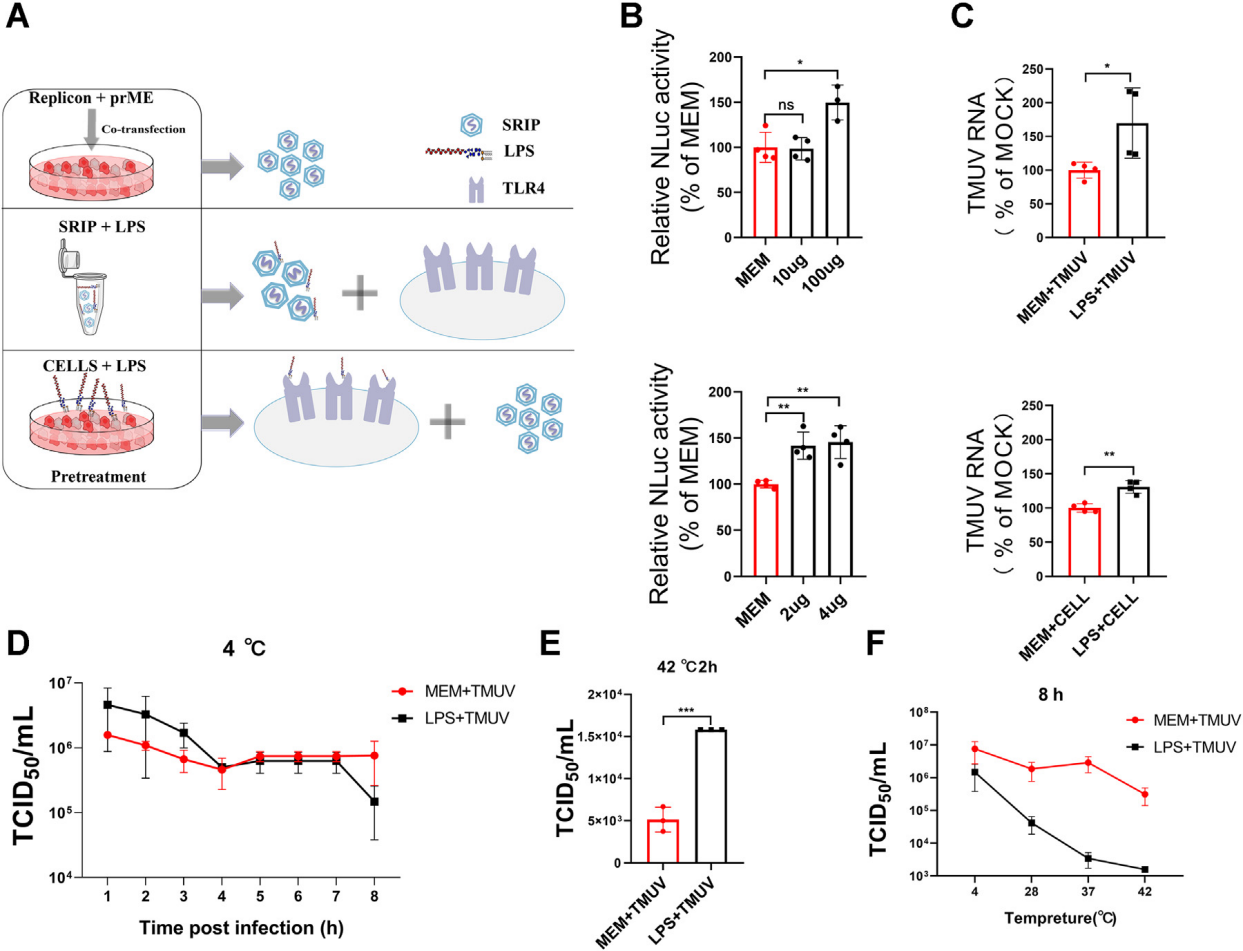
**LPS treatments increase TMUV infection in dose-dependent manner.***A*, schematic representation of the procedure used to examine the effect of LPS on SRIP infection. *Middle panel*, SRIP and LPS preincubation was performed at 4 °C for 1 h allowing virus and LPS binding; *lower panel*, LPS and duck primary cell preincubation was performed at 4 °C for 1 h allowing LPS and cell binding. At 48 h after SRIP infection, the cells were lysed to detect luminescence. *B*, Nluc activity for probes treated with LPS relative to the MEM-treated samples (average Nluc activity in these samples was taken as 100%). *Upper panel*, pretreatments of SIRP with 10 μg or 100 μg LPS; *lower panel*, pretreatment of cells with 2 μg or 4 μg of LPS. *C*, *upper panel*, TMUV virions were incubated with 20 μg of LPS or with MEM at 4 °C for 1 h and then used for infection of not treated cells; *Lower panel*, duck primary cells were incubated with 20 μg of LPS or with MEM at 37 °C for 12 h and infected with TMUV. Incubation was performed for 1 h at 37 °C after which the unbound TMUV was removed by five washes with PBS. TMUV RNA levels in obtained samples were determined using RT–qPCR, and the effect of LPS treatments was calculated relative to the MEM-treated samples. *D*, TMUV virions were incubated with MEM or 20 μg of LPS at 4 °C for the indicated times; infectivity of obtained probes was determined using limiting dilution method. *E*, TMUV virions were incubated with MEM or 20 μg of LPS at 42 °C for 2 h; infectivity of obtained probes was determined using limiting dilution method. *F*, TMUV virions were incubated with 20 μg LPS or MEM at 4, 28, 37, and 42 °C for 8 h, and infectivity of obtained probes was determined using limiting dilution method. The data are the mean ± SD of three experiments. ∗*p* < 0.05; ∗∗*p* < 0.01; ∗∗∗*p* < 0.001; and ns. LPS, lipopolysacharide; MEM, minimum essential medium; Nluc, nanoluciferase; ns, not significant; qPCR, quantitative PCR; SRIP, single-round infection virus particle; TMUV, Tembusu virus.

Next, advantage was taken from single-round infection virus particle (SRIP). These particles contain truncated TMUV genome (replicon), where region encoding for prM and E protein was replaced with that encoding nanoluciferase (Nluc) marker (21). In cells infected with SRIP, the Nluc activity level corresponds to the attachment, entry, and replication steps of TMUV life cycle. SRIPs were obtained by cotransfection of baby hamster kidney (BHK)-21 cells with the packaging plasmid prME and replicon plasmid. LPS and SRIP were mixed, incubated, and used to infect cells (Fig. 3*A*, *middle panel* and Fig. 3*B*, *upper panel*). Alternatively, SRIPs were also added to the LPS-pretreated (Fig. 3, *A*, *lower panel* and *B*, *lower panel*). Both treatments enhanced infection by SRIP up to 1.5-fold (Fig. 3*B*). First, we determine whether LPS promotes TMUV infection by increasing the attachment to the cells, LPS was incubated used to treat TMUV virions (4 °C for 1 h, Fig. 3*C*, *upper panel*) or duck primary cells (37 °C for 12 h, Fig. 3*C*, *lower panel*). The treated and untreated samples were used in infection experiment; after brief 1 h of incubation at 37 °C, the unbound TMUV was removed, and copy numbers of TMUV genomes in cell-bound virions were quantified by RT–quantitative PCR (qPCR). The results showed that both pretreatment of TMUV with LPS (Fig. 3*C*, *upper panel*) or pretreatment of cells with LPS (Fig. 3*C*, *lower panel*) significantly enhanced virus attachment.

Interestingly, we found that LPS enhanced TMUV infection only if virions were incubated with LPS at 4 °C for less than 4 h (Fig. 3*D*). The enhancement was also observed following incubation with TMUV with LPS for 2 h at 42 °C (Fig. 3*E*). After this, the enhancing effect was lost, and the TMUV infectivity was inhibited when the incubation was performed for 8 h (Fig. 3*D*). Furthermore, we found that the magnitude of inhibition, resulting from incubation of TMUV with LPS for 8 h, increased in a temperature-dependent manner (Fig. 3*F*). Combined, these results indicate that the presence of LPS is an important determinant of TMUV infection. However, its effect depends on time of exposure of TMUV virions: relatively short incubation with LPS promotes TMUV infection, whereas the prolonged incubation has an opposite effect.

### TLR4 is essential for TMUV infection

**Figure 4:**
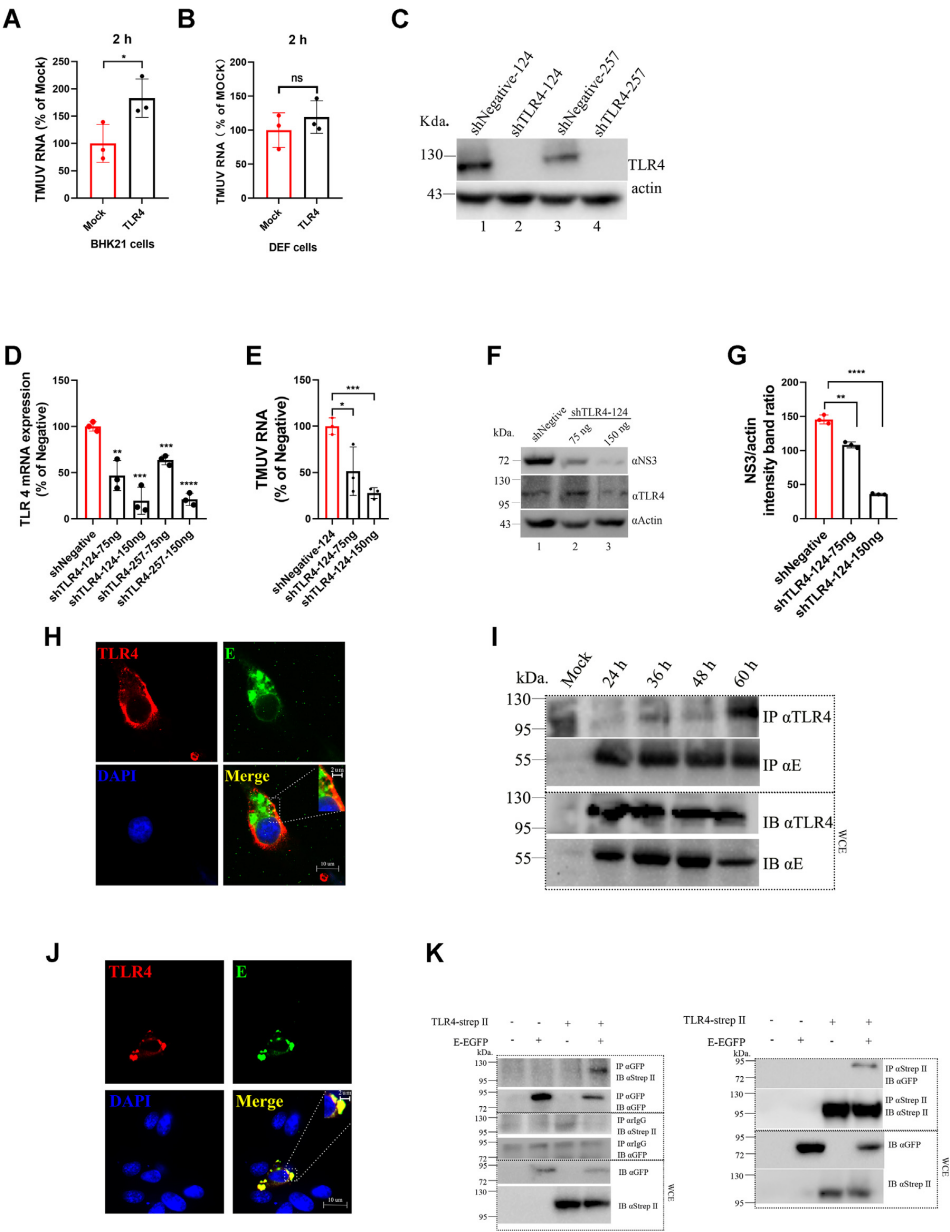
**TMUV proliferation is affected by TLR4.***A* and *B*, 1 × 10^6^ BHK-21 cells or duck primary cells were transfected with empty plasmid (mock) or plasmid expressing TLR4-strep II (TLR4). About 24 hpt, the cells were incubated with 5 × 10^5^ TCID_50_ TMUV for 2 h; the unbound TMUV was removed by washing with PBS five times. The amount of TMUV RNA in cells was determined by RT–qPCR. An average amount of RNA in mock cells was taken as 100%. *C*, BHK21 cells were cotransfected with a 75 or 150 ng of plasmid-expressing TLR4 expression plasmids and TLR4-specific shRNA (shTRL4-124 and shTLR4-257) or 150 ng of plasmid-expressing control shRNA (shNegative). Levels of TLR4 proteins were quantified by WB. *D*–*G*, 1 × 10^6^ duck primary cells were transfected with a 75 or 150 ng of plasmid-expressing TLR4-specific shRNA (shTRL4-124 and shTLR4-257) or 150 ng of plasmid-expressing control shRNA (shNegative). At 48 hpt, the cells were infected with 5 × 10^5^ TCID_50_ of TMUV. Levels of TLR4 mRNAs (*D*) and TMUV RNA genomes (*E*) were quantified by RT–qPCR at 48 hpi. Average RNA levels measured in shNegative control cells were taken as 100%. WB analysis was performed to measure the levels of TLR4 and TMUV NS3 proteins (*F*). The band intensities were measured, and a ratio of NS3 to actin band intensity is presented; an average ratio in the shNegative-transfected cells was taken as 100%. *G* and *H*, 1 × 10^6^ duck primary cells were infected with 1 × 10^5^ TCID_50_ TMUV. At 24 hpi, cells were stained with anti-E and anti-TLR4 antibodies, nuclei were counterstained with DAPI. Cells were imaged using Nikon Eclipse 80i fluorescence microscope. *I*, duck primary cells were infected with TMUV for 24, 36, 48, or 60 h, lysed in IP buffer, and TLR4 and TMUV E were immunoprecipitated using E protein antibodies (marked as IP). Obtained samples were analyzed by a WB, precipitated proteins and these in the original lysate were revealed using mouse anti-E and mouse anti-TLR4 antibodies (marked as IB). *J*, duck primary cells were cotransfected with plasmid-expressing TLR4-strep II and plasmid-expressing C-terminally EGFP-tagged TMUV E protein. Cells were fixed 24 hpt, stained with anti-strep II-tagged antibodies and DAPI; EGFP-tagged E protein was detected using EGFP fluorescence. Images were taken using Nikon Eclipse 80i microscope. *K*, duck primary cells were cotransfected with plasmids expressing TLR4-strep II and C-terminally EGFP-tagged TMUV E protein. Cells were lysed at 24 hpt, and immunoprecipitation was performed using GFP antibody (*left*) or Strep-Tactin Sepharose (*right*). Precipitated samples and original lysates were analyzed using immunoblotting with the indicated antibodies. The data on *A*–*F* are the mean ± SD of three experiments. ∗*p* < 0.05; ∗∗*p* < 0.01; ∗∗∗*p* < 0.001; ∗∗∗∗*p* <0.0001; and ns. BHK, baby hamster kidney cell; DAPI, 4′,6-diamidino-2-phenylindole; EGFP, enhanced GFP; E protein, envelope protein; hpi, hours postinfection; hpt, hours post-transfection; IP, immunoprecipitation; ns, not significant; qPCR, quantitative PCR; TCID_50_, 50% tissue culture infectious dose; TLR4, Toll-like receptor 4; TMUV, Tembusu virus; WB, Western blot.

TLR4 plays a key role in LPS-induced signal transduction (22). Studies have shown that deletions, mutations, or polymorphisms of gene encoding TLR4 as well as the downregulation of TLR4 expression all lead to hyporesponsiveness to LPS stimulation (23, 24, 25). As LPS was found to affect TMUV attachment, we speculated that TLR4 may also play a role in TMUV infection. As shown in Figure 4*A*, expression of duck TLR4 in BHK-21 cells was found to increase TMUV attachment. And expression of duck TLR4 in duck primary cells also increased TMUV attachment, but there is no significant difference (Fig. 4*B*). It might be that the transfection efficiency in primary cells is too low, results in the low expression of TLR4 protein on the surface of the cells. To verify the specificity of shRNA on TLR4 expression, we specifically silenced TLR4 expression in the other cells not expressing duck TLR4 (BHK21 cells) by cotransfection. TLR4 expression plasmids and shRNA, both designed shRNA decreased TLR4 expression significantly (Fig. 4*C*). Then, it was found that silencing of TLR4 expression (Fig. 4*D*) inhibited accumulation of viral RNA (Fig. 4*E*) and NS3 protein (Fig. 4, *F* and *G*) in duck primary cells. In conclusion, the overexpression of TLR4 promoting attachment of TMUV in cells (Fig. 4, *A* and *B*). Its depletion was similarly unfavorable for TMUV replication (Fig. 4*E*). Flaviviruses are known to utilize the E protein to interact with cell surface proteins (26) and aforementioned data confirming existence of important roles of TLR4 in TMUV entry. Here, we also examined whether the TMUV E protein may interact with TLR4. When duck primary cells were infected with TMUV, the colocalization of the E protein and endogenous TLR4 on the cell surface could be detected by immunofluorescence assay (IFA) (Fig. 4*H*). The direct interaction of these proteins was confirmed using a coimmunoprecipitation (co-IP) assay, which revealed that endogenous TLR4 was coprecipitated by TMUV E protein–specific antibody (Fig. 4*I*). Experiments using transient expression were performed to verify the specificity of TLR4 and TMUV E protein binding. When duck primary cells were cotransfected with plasmids expressing tagged versions of TLR4 and TMUV E protein, colocalization of two recombinant proteins was observed using IFA (Fig. 4*J*). Co-IP analysis showed that the tagged TMUV E protein bound TLR4 (Fig. 4*K*), confirming interaction between TLR4 and TMUV E protein and also demonstrating that this interaction is not mediated by other TMUV protein(s). Taken together, the results presented previously indicate that there is a direct interaction between TMUV and TLR4. This may indicate the positive roles TLR4 has in TMUV infection.

### TLR4 acts as a TMUV attachment factor

**Figure 5:**
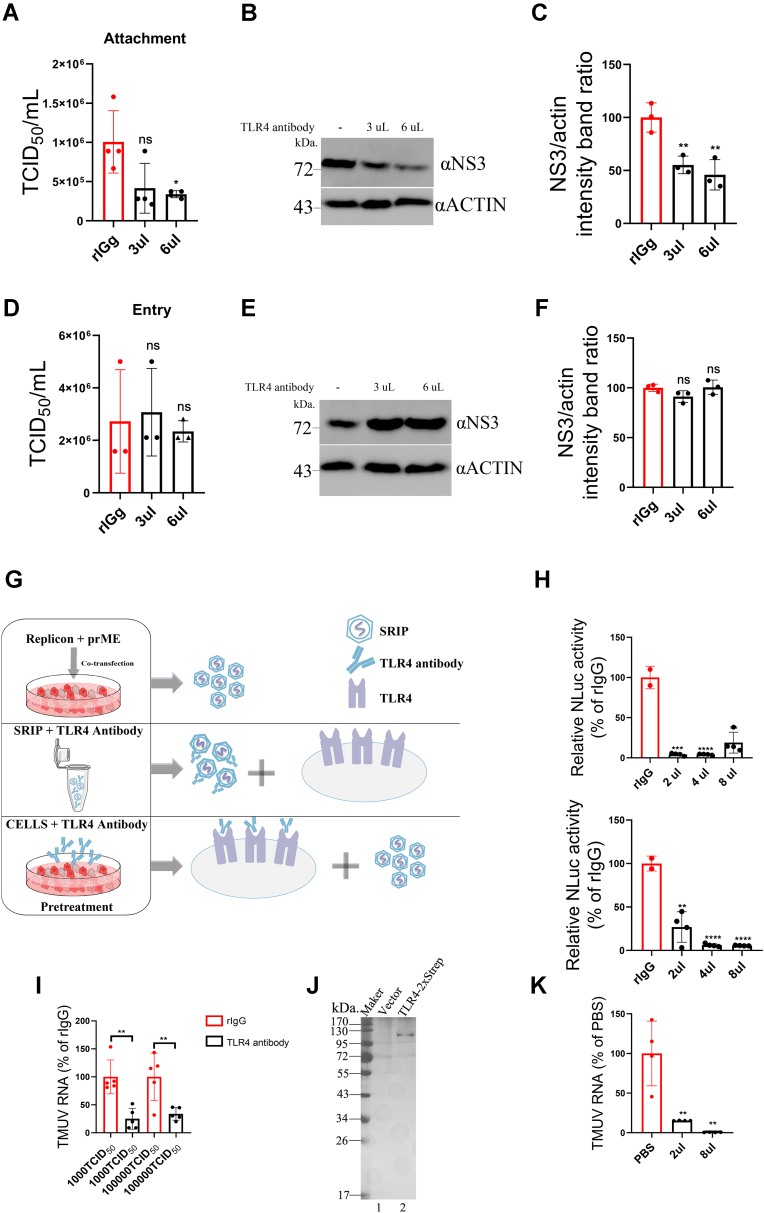
**TLR4 is vital for TMUV attachment and infection.***A*–*C*, duck primary cells were incubated with TMUV virions at 4 °C for 1 h in the absence (control) or presence of various concentrations of an anti-TLR4 antibody or normal rabbit immunoglobulin G (rIgG). The unbound TMUV was removed, cells were washed with PBS, and then incubated at 37 °C for 24 h. *D*–*F*, TMUV virions were allowed to bind to duck primary cells at 4 °C for 1 h before the addition of the anti-TLR4 antibody or rIgG. Incubation was continued at 4 °C for 1 h, after which the cells were washed with PBS. Cells were further incubated in cell culture medium containing an anti-TLR4 antibody or rIgG at 37 °C for 6 h, after which cell culture medium was replaced with fresh medium without the antibody, and incubation was continued for 18 h at 37 °C. *A* and *D*, TMUV titers in the supernatants detected by limiting dilution method. *B* and *E*, expression of NS3 in the TMUV-infected cells were detected by a Western blot analysis, “-” designates the presence of rIgG. *C* and *F*, quantification of NS3 expression relative to actin. Average ratio at the presence of rIgG was taken as 100%. The mean values ± SD of data from three independent experiments are shown in *A*, *C*, *D*, and *F*. *G*, schematic representation of the procedure used to examine the effect of the anti-TLR4 antibody on infectivity of TMUV SRIP. *Upper panel*, the generation of SRIP; *middle panel*, SRIPs were preincubated with anti-TLR4 antibody at 4 °C for 1 h; *lower panel*, duck primary cells were preincubated with anti-TLR4 antibody at 4 °C for 1 h. SRIP attachment assays were performed as described for Figure 3. *H*, SRIPs treated with antibodies were used to infect duck primary cells (*upper panel*), or duck primary cells preincubated with antibodies were infected with SRIP (*lower panel*). Infected cells were incubated for 48 h; after this, the cells were lysed, and NLuc activities were measured. NLuc activities in cells infected with rIgG-treated SRIP (*upper panel*) or in cells pretreated with rIgG (*lower panel*) were taken as 100%. *I*, 3 μl of anti-TLR4 antibody or rIgG were incubated with 1 × 10^3^ or 1 × 10^6^ TCID_50_ TMUV at 4 °C for 1 h. The attachment assay was performed using treated samples and duck primary cells, after which cells were washed with PBS. The copy number RNA genomes present in TMUV virions bound to cells were determined by RT–qPCR. TMUV RNA copy numbers detected for probes incubated with rIgG-treated samples were taken as 100% for each of the used viral doses. *J*, 5 μl of purified recombinant TLR4 protein was analyzed using SDS-PAGE, the gel was stained with *silver*. *K*, 2 μl or 8 μl of TLR4 protein (from the same stock analyzed on *J*) was incubated with 5 × 10^5^ TCID_50_ of TMUV at 4 °C for 1 h. Treated samples were used to infect duck primary cells for 1 h. The unbound TMUV virions were removed by washing with PBS, and the copy number of RNA genomes present in TMUV virions bound to cells were determined by RT–qPCR. RNA copy number in the control sample where TMUV virions were treated with PBS was taken as 100%. The data on *A*, *C*, *D*, *F*, *H*, *I*, and *K* are the mean ± SD of at least three experiments. ∗∗*p* < 0.01; ∗∗∗∗*p* <0.0001; ns. NLuc, nanoluciferase; ns, not significant; qPCR, quantitative PCR; SRIP, single-round infection virus particle; TCID_50_, 50% tissue culture infectious dose; TLR4, Toll-like receptor 4; TMUV, Tembusu virus.

To further clarify the role of TLR4 in the TMUV infection, anti-TLR4 antibody and purified TLR4 protein were used to analyze. In the first experiment, duck primary cells were incubated with TMUV at 4 °C for 1 h, a condition that allows virus attachment but not cell entry, in the presence of an anti-TLR4 antibody; normal (unspecific) rabbit immunoglobulin G was used as negative control. After 24 h of incubation at 37 °C, the titers of TMUV were determined by a serial dilution method (Fig. 5*A*), and the expression of NS3 in infected cells was analyzed using Western blot (Fig. 5, *B* and *C*). It was observed that the treatment with TLR4 antibody reduced TMUV infection: 60% reduction in released infectious virus titer (Fig. 5*A*) and statistically significant nearly 70% reduction in the NS3 protein level (Fig. 5*C*) were observed. In contrast, when antibodies were added after attachment step, the TLR4 antibody failed to inhibit TMUV infection (Fig. 5, *D*–*F*). These results imply that TLR4 is involved in the attachment step of TMUV virions. Second, we determined the inhibitory activity of the TLR4 antibody using TMUV SRIP infection model (Fig. 5*G*). The pretreatment of SRIP (Fig. 5*G*, *middle panel* and Fig. 5*H*, *upper panel*) or duck primary cells (Fig. 5, *G* and *H*, *lower panels*) with an anti-TLR4 antibody for 1 h at 4 °C significantly inhibited SRIP infection; up to 95% reduction of infection was observed for both treatments.

To further confirm that the TLR4 antibody impaired TMUV attachment, duck primary cells were incubated at 4 °C for 1 h with low- or high-dose TMUV virions that were pretreated with the TLR4 antibody or negative control antibody (rabbit immunoglobulin G). Again, it was observed that treatments with the TLR4 antibody inhibited virus attachment; the effect was prominent and significant both at case of high and low dose of TMUV (Fig. 5*I*). Finally, to explore the possible interaction between TLR4 and TMUV, we expressed and affinity purified recombinant duck TLR4 protein (Fig. 5*J*). About 2 μl or 8 μl of obtained TLR4 protein stock was used to incubate with TMUV particles at 4 °C for 1 h. Subsequent binding assay performed with duck primary cells, which revealed that treatment with recombinant TLR4 markedly blocked TMUV binding to duck primary cells; compared with PBS-treated (used as negative control) samples, the attachment was reduced, depending on amount of used TLR4 protein, from 75% to 99% (Fig. 5*K*). Collectively, data from these experiments confirm that TLR4 functions as important factor for TMUV attachment to duck cells.

### TLR4 is essential for LPS-enhanced TMUV adsorption

**Figure 6:**
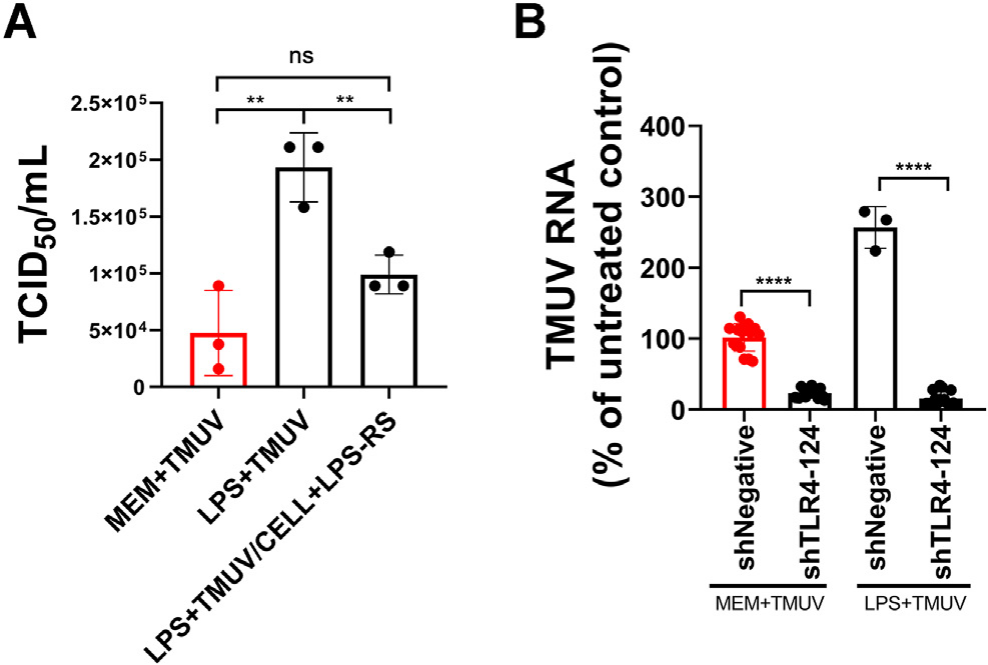
**TLR4 is required for LPS-enhanced TMUV attachment.***A*, 1 × 10^5^ TCID_50_ TMUV virions were preincubated with 20 μg of LPS (LPS + TMUV) or MEM (MEM + TMUV) for 1 h at 4 °C and used for infection of duck primary cells at 4 °C for 1 h. In addition, part of duck primary cells were pretreated with the 20 μg TLR4 inhibitor LPS-RS at 37 °C for 1 h and infected with LPS + TMUV (LPS + TMUV/CELL + LPS-RS) at 4 °C for 1 h. All infected cells were washed with PBS and then incubated at 37 °C. At 24 hpi, supernatants were harvested, and the amount of infectious virus was determined using limiting dilution assay. *B*, duck primary cells grown in 12-well plates were transfected with 2 μg of shRNA-expressing plasmid shTLR4-124 or shNegative. At 48 hpt, TMUV preincubated with 20 μg LPS for 1 h at 4 °C (LPS + TMUV) or TMUV preincubated with MEM (MEM + TMUV) was added, and cells were incubated for 1 h at 4 °C. The unbound TMUV virions were removed by washing with PBS, and the copy number of RNA genomes present in TMUV bound to cells was determined by RT–qPCR; RNA copy number in cells transfected with shNegative and infected with MEM + TMUV (untreated control) was taken as 100%. The data are the mean ± SD of three experiments. ∗∗*p* < 0.01; ∗∗∗∗*p* < 0.0001; and ns. hpi, hours postinfection; hpt, hours post-transfection; LPS, lipopolysaccharide; MEM, minimum essential medium; ns, not significant; qPCR, quantitative PCR; TCID_50_, 50% tissue culture infectious dose; TLR4, Toll-like receptor 4; TMUV, Tembusu virus.

As both LPS and TLR4 were found to be involved in TMUV attachment (Figure 2, Figure 3, Figure 5), we hypothesized that LPS may enhance the interaction strength between TMUV E protein and TLR4. TLR4-mediated LPS recognition requires the participation of protein called MD2; coherently, the MD2 gene-deficient mice are tolerant to LPS treatment (27). MD2 forms TLR4-MD2 heterodimers that are used as the LPS signal receptors on macrophages. This mode of action opened possibility to treat cells with TLR4 antagonists (LPS-RS), which can compete with LPS for MD2 binding and thus inhibit TLR4 signaling (28, 29). Here, we incubated TMUV with LPS prior and added it into duck primary cells either treated or not with TLR4 antagonist (LPS-RS). It was found that the treatment of cells with LPS-RS significantly reduced positive effect of LPS on TMUV infection; the observed inhibition was approximately twofold (Fig. 6*A*). This finding was further confirmed using TLR4 knockdown assays: suppression of TLR4 levels inhibited TMUV attachment and, importantly, all gain resulting from LPS treatment of TMUV virions was lost (Fig. 6*B*). These results clearly demonstrate that TLR4 is involved in LPS-enhanced TMUV adsorption to the duck cells.

### TMUV inhibits LPS-mediated interleukin 6 induction

**Figure 7:**
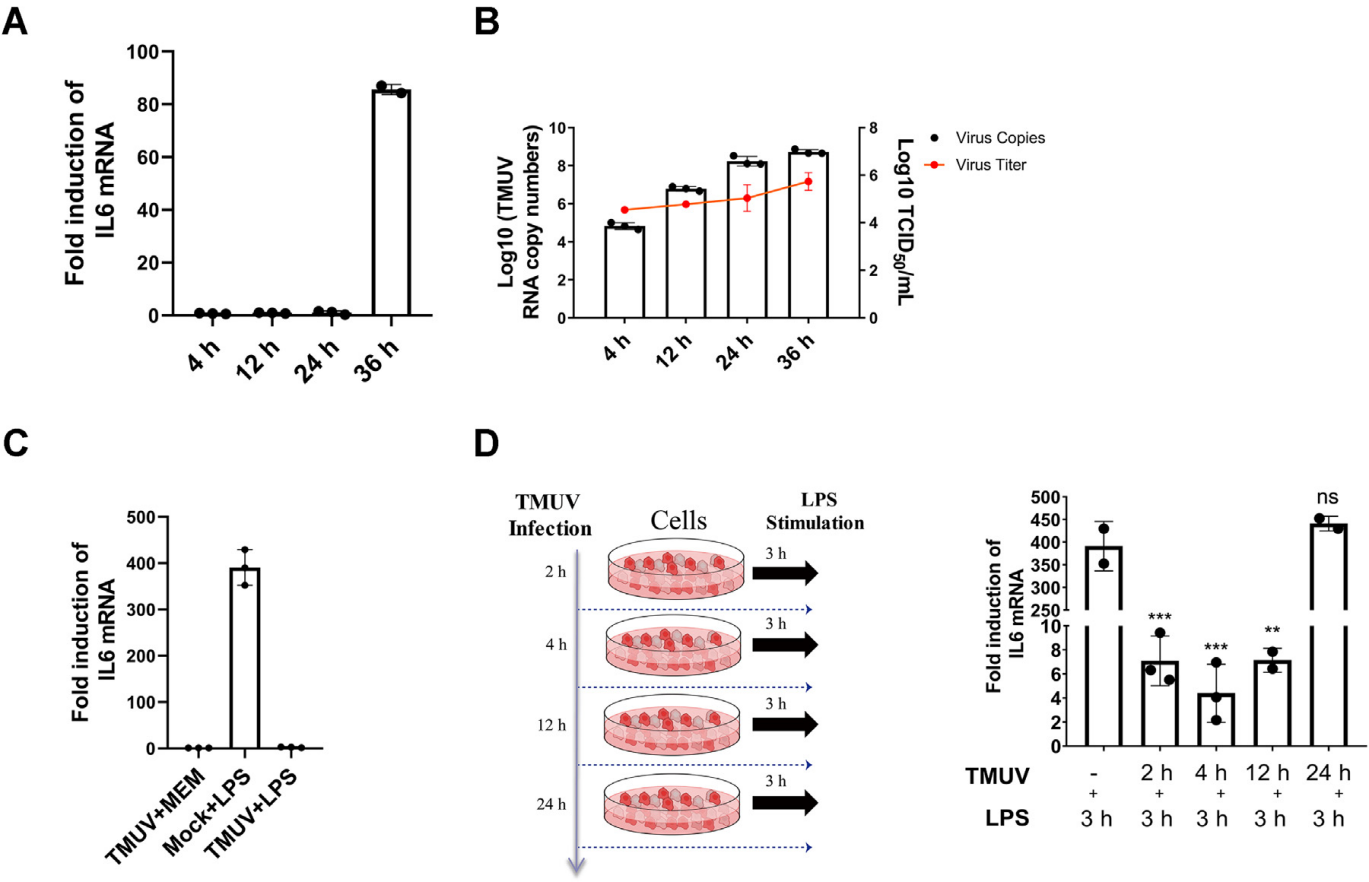
**TMUV infection impairs LPS-induced IL6 production.***A* and *B*, 1 × 10^6^ duck primary cells were grown in a 12-well plate and infected with 1 × 10^5^ TCID_50_ TMUV for 4, 12, 24, and 36 h. At these time points, cells were collected, total RNA was isolated, and the expression of IL6 mRNA (*A*) and intracellular copy number of TMUV RNA genome (*B*) were analyzed by RT–qPCR. Culture supernatants were also collected and assayed for extracellular infectious virus production using limiting dilution assay (*B*). *C*, duck primary cells were infected with 5 × 10^5^ TCID_50_ of TMUV and treated with LPS (TMUV + LPS) or not treated (TMUV + MEM). In addition, control cells were mock infected and treated with LPS (mock + LPS). Infected and/or LPS-treated cells were incubated for 9 h at 37 °C. *D*, duck primary cells were mock infected or infected with 5 × 10^5^ TCID_50_ of TMUV for 2, 4, 12, and 24 h. Then, the cells were stimulated with 20 μg LPS for 3 h. *C* and *D*, after indicated incubation times, cells were collected, total RNA isolated, and the levels of IL6 mRNA were measured by specific RT–qPCR. The data are the mean ± SD of three experiments. For *A*, *C*, and *D*, the IL6 mRNA levels in infected and/or LPS-treated cells are shown relative to the mock-infected nontreated cells (IL6 mRNA levels in these cells were taken as 1). ∗∗*p* < 0.01; ∗∗∗*p* < 0.001; ns. IL6, interleukin 6; LPS, lipopolysaccharide; MEM, minimum essential medium; ns, not significant; qPCR, quantitative PCR; TCID_50_, 50% tissue culture infectious dose; TMUV, Tembusu virus.

To further analyze the functional implications of interaction between LPS, TLR4, and TMUV, duck primary cells were infected with TMUV for 4, 12, 24, or 36 h. And interleukin 6 (IL6) mRNA and viral genome RNA copy numbers were determined. It was found that at 4, 12, and 24 h, IL6 mRNA levels in TMUV-infected cells were very low (Fig. 7*A*) and comparable with these in mock-infected cells. In contrast, TMUV RNA copy number increased approximately 2500 times from 4 to 24 h (Fig. 7*B*). The massive replication of TMUV did not cause the induction of IL6 mRNA expression. Thus, during active stages of TMUV RNA replication, the induction of IL6 mRNA expression was likely actively suppressed by the virus. The expression of IL6 can be induced by various stimuli, including LPS treatment (30). Indeed, we observed that LPS treatment of cells for 9 h increased IL6 mRNA levels nearly 400 times; interestingly, this effect was completely lost when cells were infected with TMUV prior (Fig. 7*C*). The results indicate that TMUV infection suppresses the ability of LPS to induce IL6 production. Finally, we also investigated whether TMUV infection inhibits the LPS-mediated induction of IL6 expression in a time-dependent manner (Fig. 7*D*). The results revealed that at the early stage of infection, TMUV inhibited LPS-induced IL6 production; the inhibition was roughly at similar level for 2, 4, and 12 h p.i. but was lost at 24 h p.i. (Fig. 7*D*). These results clearly show that at early stages of infection, TMUV can suppress IL6 expression. The mechanism of this suppression remains to be revealed although it can be speculated that TMUV and/or TMUV E protein might occupy the binding sites of LPS on the cell surface, which temporarily (during stages of rapid virus genome replication) leads to inhibition of TLR4-mediated signal transduction.

## Discussion

DENV infection of bone marrow–derived macrophages results in rapid induction of the transcription of inflammation-related factors (16, 31). In contrast, we found that at the early stage, TMUV infection of duck primary cells did not induce IL6 production, which become activated only at 24 hpi (Fig. 4*C*) or even later (Fig. 7*A*). Furthermore, cells infected with TMUV for 2 to 12 h were resistant to the stimulation of LPS, a strong inducer of IL6 expression, adding of which could not induce the high level production of IL6 (Fig. 7*D*). Based on these data, it was assumed that the virus interacted with IL6-related receptors, leading to active inhibition of the production of inflammatory factors at the early stage of infection. Because LPS cannot directly enter the cell, the cell surface receptor such as TLR4 is needed to transduce the LPS signal (32). Therefore, we speculate that LPS competes with TMUV virions or TMUV-encoded protein(s) for binding to the same cell receptor. This assumption is supported by findings from colocalization and co-IP experiments, which showed that TLR4 could interact with the E protein of TMUV (Fig. 4, *I*–*L*). TLR4 plays a complex role in the viral life cycle through different mechanisms. The G protein of vesicular stomatitis virus, F protein of respiratory syncytial virus, and glycoproteins of Ebola virus (33) can interact with TLR4 when these viruses infect cells resulting in activation of TLR4 signaling and induction of the production of downstream inflammatory factors. The knockdown of TLR4 expression in Kaposi sarcoma herpesvirus–infected cells decreased the expression of inflammatory factors and interferon genes and resulted in a higher viral gene expression (34). Even more complicated, viruses from different families infect TLR4 knockout mice, resulting in a mortality rate similar to that of the control group (35, 36, 37, 38).

Interestingly, we observed a massive increase in IL6 expression in TMUV-infected duck primary cells at 36 hpi (Fig. 7*A*). This phenomenon suggests an existence of competition between TMUV infection and activation of innate immune response. Cells employ pattern recognition receptors to sense viruses, and viruses use multiple strategies to escape cell sensing. Some factors involved in the flavivirus life cycle, such as NS1, have been shown to induce the production of IL6 (36, 39, 40). Our results suggest that the E protein of TMUV plays an important and opposite role by acting as an inhibitor of IL6 production. This E protein could counteract induction of cytokine production induced by other factors, such as NS1, and facilitating virus proliferation at early stages of infection. It is plausible that at the late stage of the infection, E protein cannot maintain this function, possibly because of the high levels of NS1 expression and secretion. Therefore, the massive increase in IL6 expression occurs at 36 hpi (Fig. 7*A*); an ability of TMUV infection to counteract IL6 induction by external inducers (such as LPS) is also lost at later time points (Fig. 7*D*). On mechanistic side, it is also possible that earlier in the infection, the amount of TMUV is too low to activate the TLR4 signaling pathway. Higher TMUV load may lead to a higher expression of other viral proteins, such as NS1, which can induce the production of IL6. There is also likely a synergistic action between these TMUV-encoded components and potent IL6 inducer such as LPS; the presence of both enables cells to reach the tipping point faster (compare Fig. 7, *A* and *D*). It is interesting. However, that induction of IL6 production occurs at the time when virus RNA copies have already reached high levels (Fig. 7*B*). This is unlikely coincidental and most likely indicates that at this stage of infection, IL6 production causes little to no harm for virus, and therefore, suppression of IL6 production becomes not essential.

**Figure 8:**
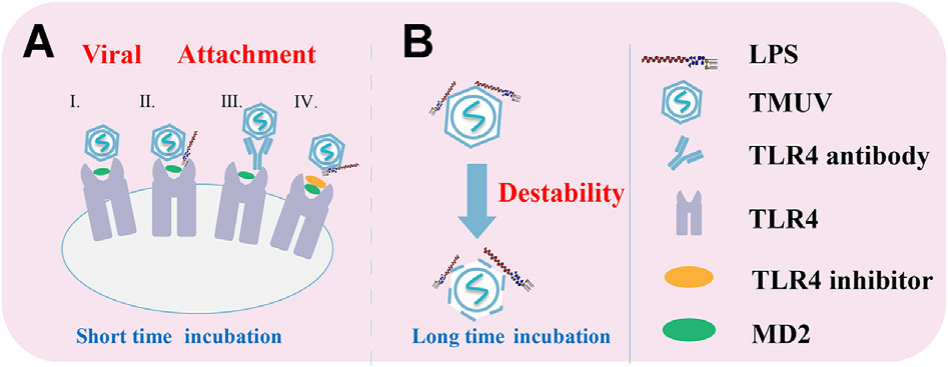
**Proposed model of TMUV entry into duck primary cells.***A*, TLR4 is involved in the attachment steps of TMUV infection cycle and can function in the absence of LPS. However, the presence of LPS can significantly enhance the ability of TMUV virions for attachment to duck primary cells. Enhancing occurs as a result of short-term incubation of TMUV virions with LPS and can be affected by factors shown in *B*. Long-term incubation of TMUV virions with LPS reduces the stability of the virions, and their ability to infect duck primary cells is diminished. LPS, lipopolysaccharide; TLR4, Toll-like receptor 4; TMUV, Tembusu virus.

Because TMUV can also inhibit LPS-induced IL6 production and LPS is an agonist of TLR4, we further explored the interaction between LPS and TMUV. Previous data suggest that poliovirus infections are enhanced by LPS, which enhances stability of poliovirus virions and increases their bleach resistance (10, 41). Here, we observed that pretreatment of duck primary cells with LPS also did enhance TMUV infection and that pretreatment of TMUV virions with LPS resulted in similar effect (Figs. 2, and 3). Unlike to the mechanism promoting enterovirus infection, we found that LPS promotes virus TMUV infection by increasing its attachment to the cells and that this effect is lost when time of treatment of TMUV virions with LPS is prolonged (Figs. 3*D* and 8).

Attachment of virions to host cells is a complex process, and many molecules of the host cell are involved in the entry of viruses into cells. The virion envelope can also be embedded with host molecules that participate in the virus life cycle (17, 42, 43). DENV incorporates phosphatidylserine into its membrane to promote TIM- and TAM-mediated DENV infection (42). TLR4-mediated LPS recognition requires the participation of MD2 (27), a secreted glycoprotein that lacks a transmembrane structure. The binding of LPS to CD14, another coreceptor for complex involved in LPS recognition, is mediated by LPS-binding protein (LBP) (44). Thus, LPS invades the host, binds the cell surface with the help of LBP/CD14, and then acts on TLR4 with the help of MD2. This results in signaling that activates the nuclear NF-κB and mitogen-activated protein kinase signaling pathways, thereby promoting the activation of the expression of various inflammatory cytokine genes (45). Further research can explore whether cellular molecules that bind LPS, such as LBP, TLR4, MD2, or CD14, are present in the TMUV membrane. Studies have shown that there is also a receptor system consisting form multiple protein molecules (including CD14, TLR4, CXCR4, HSP70, HSP90, GDF5, etc.) on the cell surface (46). LPS binds cells through this receptor system and participates in further signal transduction. The interaction between TMUV and that LPS receptor cluster is unknown. Given the complexity and importance of the system used for LPS recognition and signaling, it is remarkable that TMUV has an ability to use the complex (or, at the very least several of its components) to promote attachment of its virions to the cells without triggering antipathogen responses. This phenomenon warrants further studies that can promote our understanding about not only TMUV infection but also functioning of LPS receptor clusters.

Interestingly, we found that the incubation of TMUV virions with LPS at 4 °C beyond 4 h seriously affected the infectivity of the virus. At higher temperatures, the negative impact of long-term incubation increased (Fig. 3*F*), but, importantly, beneficial effect of a short incubation time was maintained even at 42 °C (Fig. 3*E*). Notably, the body temperature of normal ducks is approximately 42 °C making these findings relevant to *in vivo* infection. Indeed, we observed that reducing intestinal flora affected TMUV infection when ducks were infected with gavage. The intestinal flora produces a large amount of LPS, which most likely supported TMUV infection.

Collectively, our study identified LPS as novel facilitator of TMUV infection both *in vitro* and *in vivo*. Furthermore, our study shed light not only on the sophisticated TLR4-mediated effect on TMUV infection that was not limited to innate immune responses launched against virus invasion but also involved important role of TLR4 in TMUV attachment. These findings expand our knowledge of the role of commensal microbiota in regulating immunity in the intestinal mucosa during flavivirus infection. The oral transmission of TMUV was experimentally demonstrated. This phenomenon, currently rather unique for normally vector-transmitted viruses such as flaviviruses, warrants additional detailed studies that are currently hampered by the lack of required models. For example, TLR4 gene knockout ducks are needed for further studying the role of TLR4 and LPS in oral TMUV infection, which may expand our understanding of the nonvector transmission of TMUV in greater detail. We consider such studies highly important both from academic and practical points of view. As demonstrated here, such studies have a potential to result in discovery of novel roles of host factors and to reveal complex interactions between host, virus, and microbiota. It is also clear that existence of novel (or alternative) modes of transmission may significantly increase potential of viruses to spread, and their understanding is therefore important to prevent or limit potential virus outbreaks.

## Experimental procedures

### Cells and virus

BHK-21 (American Type Culture Collection; CCL-10) cells were grown in Dulbecco's modified Eagle's medium (Sigma–Aldrich) containing 10% fetal bovine serum. The duck primary cells used in this study were duck embryonic fibroblasts obtained from 10-day-old duck embryos and propagated in Dulbecco's modified Eagle's medium supplemented with 10% newborn calf serum (Life Technologies). All cells were cultured in an incubator at 37 °C with 5% CO_2_. The duck TMUV strain CQW1 (GenBank accession number: KM233707.1) has been previously reported (47); the titer of used TMUV stock was 10^7^ TCID_50_/ml.

### SRIP preparation

SRIPs were prepared as previous described (21). Briefly, BHK21 cells in a 12-well plate grown to 70 to 90% confluence were cotransfected with equal amounts of mC-Replicon-NLuc and pCDNA3.1-C16prME plasmids. After transfection, the cells were incubated at 37 °C with 5% CO_2_ for 4 days. The TMUV SRIP-containing supernatants were harvested every day, aliquoted, and stored at −80 °C.

### Antibodies

The antibodies against β-actin, GFP, strep II, and TLR4 purchased from ABclonal, and mouse anti-TMUV E monoclonal antibody (48) was used for immunoblotting experiments. The mouse polyclonal antibodies against NS3 of TMUV were generated in house. The concentration of TLR4 antibody is 0.97 mg/ml.

### Duck treatments

SPF duck embryos were purchased from Harbin Veterinary Research Institute. The ducklings were maintained in sterile isolators at Sichuan Agricultural University, ethical board of which approved all procedures. One-day-old SPF ducklings received an intragastric gavage of 500 μl of a mixture of the following Abxs at day 0: ampicillin (0.5 mg/ml), gentamicin (0.5 mg/ml), metronidazole (0.5 mg/ml), and neomycin (0.5 mg/ml) (all purchased from Sigma–Aldrich); then, all Abx concentrations were increased to 1 mg/ml at day 5; Abx concentrations were increased to 2 mg/ml at day 10; and Abx concentrations were increased to 10 mg/ml at day 15. The ducks were infected with 1 × 10^6^ TCID_50_ TMUV at day 20. Thus, in total, the ducks were treated with Abx for a total of 20 days. A periodic bacteriologic examination of feces was used to evaluate the efficacy of the Abx-treatment protocol. Fresh fecal samples were collected from the ducks after the 20th day from beginning of treatment, homogenized, plated on LB agar plates with 10% sheep blood, and incubated under anaerobic conditions at 37 °C for 2 days, followed by incubation under aerobic conditions at 37 °C for 1 day.

For the intestinal transit time assays, the ducks were gavaged with 2 ml of 8% Evans blue dye (Selleck) after treatment of Abx at 20th day. Fecal samples were collected at 4, 12, and 24 h, weighed, and resuspended in 0.1 g/ml sterilized PBS. The presence of dye was determined by reading the absorbance at 626 nm with a Multiskan Spectrum (Thermo Fisher Scientific).

### Cell infection, RNA extraction, and RT–qPCR

Duck primary cells were infected with TMUV. At the indicated times, the total RNA was extracted using RNAiso plus reagent (Takara). The RNA was reverse transcribed with HiScript III-RT SuperMix (Vazyme) using random hexamer primers. The expression levels of the IL6, IFNbeta, OAS, and PKR genes were then quantified using the SYBR Green method (Abm) and a LightCycler 480 System instrument (Roche); mRNA copy numbers of these genes were normalized to the copy number of mRNA β-actin gene. The TMUV RNA copy numbers were detected by absolute qPCR according to the real-time qPCR procedure previously established in our laboratory (49).

### RNAi-mediated silencing of TLR4 expression

The RNAi constructs were designed using Thermo Fisher RNAi target finder (http://rnaidesigner.thermofisher.com/rnaiexpress/). Sequences GCAACCTTCTATGGTTTAACACGAATGTTAAACCATAGAAGGTTGC and GCTACAGGTCAACAGACTAATCGAAATTAGTCTGTTGACCTGTAGC were constructed from oligonucleotides and cloned into the shRNA expression vector pGPU6-GFP-Neo, resulting in construct designated as shTLR4-124 and shTLR4-257, respectively. Plasmid designated as shNegative-124 (GCTTCCTACTATGGTAAAACACGAATGTTAAACCATAGAAGGTTGC) and shNegative-127 (GCAACAAATCAACTGACCAAGCGAAATTAGTCTGTTGACCTGTAGC) were constructed as negative control.

### Immunoblot analysis

Cells were cultured in 12-well plates and harvested with lysis buffer containing a cocktail of protease and phosphatase inhibitors (Thermo Fisher Scientific). Equal amounts of the samples were then separated by SDS-PAGE using 10% polyacrylamide gels. Proteins were transferred to polyvinylidene difluoride membranes, incubated with appropriate primary and secondary antibodies, and visualized using an enhanced chemiluminescence system (Bio-Rad). The expression of β-actin, which was used as a loading control, was detected with mouse anti-β-actin monoclonal antibody (Transgen Biotech).

### IP assays

The IP assays were performed as previously described (49). Briefly, cells were cultured in 60 mm plates and harvested by lysis in IP buffer (50 mM Tris–HCl [pH 7.4], 150 mM NaCl, and 1 mM EDTA supplemented with a protease inhibitor cocktail). A portion of the total lysates was retained as a whole-cell extract. For each IP reaction, 0.9 ml of the cell lysate was incubated with 30 μl of protein G-sepharose suspension (GE Healthcare) and 0.5 μg of antibody at 4 °C for 3 h. The protein G-sepharose beads were intensively washed with lysis buffer containing 0.5 M NaCl. The precipitates were then denatured, and obtained samples were subjected to SDS-PAGE and an immunoblot analysis.

### Indirect IFAs

Duck primary cells or BHK-21 cells were seeded on coverslips that were placed in 6-well plates. When the cells reached approximately 70 to 90% confluence, they were cotransfected with the plasmids expressing duck FLAG-tagged TLR4 and TMUV E protein fused with enhanced GFP. At 24 h post-transfection, the cells were fixed in 4% paraformaldehyde for 1 h and permeabilized with 0.25% Triton X-100 for 1 h at 4 °C. After three washes with PBS, the cells were blocked with 5% bovine serum albumin in PBS for 1 h and then incubated with the mouse anti-FLAG primary antibodies, washed, incubated with the secondary antibodies, washed again, and visualized under a Nikon 80i microscope using magnification 600×.

### Recombinant TLR4 expression in mammalian cells and affinity purification

The full-length coding sequence of duck TLR4 gene was amplified and fused with a sequence encoding for C-terminal strep II tag using overlapping PCR. The obtained sequence encoding TLR4-strep II tag sequence was inserted into a *pCAGGS* vector; for some experiments, the strep II tag was replaced with FLAG tag. The obtained plasmid was used in aforementioned transfection and cotransfection experiments.

To obtain recombinant TLR4 protein, human embryonic kidney 293T cells were transfected with plasmid expressing TLR4 strep II using Lipofectamine 3000 (Thermo Fisher Scientific). The cells were harvested 48 h post-transfection and broken using three freeze–thaw cycles. The obtained sample was centrifuged at 12,000*g* for 10 min to remove cell debris. The TLR4-strep II protein was purified from obtained supernatant using Strep-Tactin Sepharose according to the manufacturer’s (IBA) instructions.

### Statistical analysis

Statistical analyses were performed, and graphical images were prepared using Prism 8 software (GraphPad Software, Inc). Unless otherwise stated, the results are shown as the means ± SD of three independent experiments, and statistical significance was calculated using Student's *t* test for pairwise comparisons. The statistically significant differences are indicated as follows: ∗*p* < 0.05; ∗∗*p* < 0.01; ∗∗∗*p* < 0.001; ∗∗∗∗*p* < 0.0001; and ns, not significant.

## Data availability

Raw mass spectrometry data will be available upon request.

## Conflict of interest

The authors declare that they have no conflicts of interest with the contents of this article.
